# A Prospective Study on the Suprapatellar Approach for Tibial Shaft Fractures: Insights Into Functional and Radiological Outcomes

**DOI:** 10.7759/cureus.74862

**Published:** 2024-11-30

**Authors:** Surya Malasani, Gaurav Jha, Sai Ganesh

**Affiliations:** 1 Orthopedics and Traumatology, University Hospitals of Leicester, Leicester, GBR; 2 Trauma and Orthopedics, Leicester Royal Infirmary, Leicester, GBR; 3 Orthopedics and Traumatology, KIMS - Saveera Hospital, Anantapur, IND

**Keywords:** fracture healing, functional outcomes, intramedullary tibial nailing, suprapatellar nailing, tibial locking nailing, tibial shaft fractures

## Abstract

Background: Tibial shaft fractures are among the most common long bone injuries and often can be challenging to manage surgically. While infrapatellar (IP) intramedullary nailing (IMN) has been a widely accepted treatment, its limitations have led to the emergence of alternative approaches, such as suprapatellar nailing (SPN) in a semi-extended knee position.

Aim: To evaluate the clinical, radiological, and functional outcomes of tibial shaft fractures treated with an SPN approach in a semi-extended knee position.

Methods: A prospective study was conducted at Saveetha Medical College, including 20 patients diagnosed with tibial shaft fractures between August 2021 and December 2022. All patients underwent IMN using a suprapatellar approach after being assessed for surgical fitness and providing informed consent. Clinical and radiological follow-ups were performed over a 12-month period to evaluate knee range of motion, fracture healing, implant positioning, and knee pain, using the Lower Extremity Functional Scale (LEFS) and Visual Analog Scale (VAS).

Results: The study cohort consisted of 20 patients, with a male-to-female ratio of 3:1. The majority (70%) were aged between 20 and 40 years. Fractures affected the left tibia in 11 cases and the right in nine cases. Among the fractures, 12 were closed and eight were open, classified according to the Gustilo-Anderson classification as ranging from Grade 1 to Grade 3. The mean surgery duration was 83.5 minutes, with an average blood loss of less than 100 mL. The mean time to fracture union was 12 weeks, and the mean LEFS score recorded was 75.75. Three patients experienced complications, which were managed successfully. Overall, patients demonstrated favorable clinical, radiological, and functional outcomes with minimal knee pain post-surgery.

Conclusion: Suprapatellar IMN in the semi-extended position is a viable and effective surgical approach for managing tibial shaft fractures, providing good clinical, radiological, and functional outcomes with minimal complications.

## Introduction

Tibial shaft fractures are among the most common long bone fractures, accounting for approximately 2% of all fractures in adults [1,2]. Tibial shaft fractures have an incidence of 16.9 per 100,000 annually, with a distinct bimodal age distribution, peaking in young adults around 20 years old, often due to high-energy trauma such as motor vehicle accidents, and in older adults near 50 years of age, typically resulting from low-energy falls [2-4]. The tibia's subcutaneous position and limited soft tissue coverage present unique challenges in the management of these fractures, influencing both treatment choice and outcomes.

Management options for tibial shaft fractures include both conservative and surgical approaches. Surgical treatments range from plating and internal fixation to external fixation [5]. Among these, intramedullary nailing (IMN) has long been considered the gold standard due to its ability to achieve stable fixation, maintain fracture alignment, and facilitate early mobilization [6]. Traditionally, the infrapatellar (IP) approach for IMN has been favored because it is minimally invasive and supports early functional rehabilitation​ [7]. However, this method has inherent challenges. The technical difficulty of achieving and maintaining proper reduction during surgery is exacerbated by the force exerted by the quadriceps muscle, which can lead to displacement of the proximal fracture fragments when the knee is in a flexed position. This may increase the risk of valgus and procurvatum deformities, contributing to the potential for malunion [7,8]. Additionally, postoperative anterior knee pain is a significant concern following IMN insertion through the IP approach, with reported incidence rates ranging from 10% to 80%​ [9].

Recent advances in nail design and reduction techniques have expanded the indications for IMN to include both proximal and distal tibial fractures, particularly those involving the metaphyseal region. Establishing an anatomic starting point is critical for ensuring successful outcomes in these cases [10]. The suprapatellar nail (SPN) approach, performed with the knee in a semi-extended position, has gained attention as a promising alternative to the traditional IP method. This approach addresses some of the limitations of the IP technique by allowing for better fracture reduction, improved alignment, and reduced radiation exposure and operative time [10,11]. The semi-extended position also reduces the risk of complications associated with knee hyperflexion, such as malalignment [9]. Cole et al. introduced the suprapatellar approach using a midline quadriceps tendon entry site, which is performed with the knee in a semi-extended position​ [12]. This technique facilitates the proper reduction of fractures, particularly those with apex anterior deformities, and improves the ease of intraoperative imaging [13]. Despite some concerns that the suprapatellar approach could lead to intra-articular damage or cartilage injury, these risks have not been conclusively demonstrated.

The present study aims to analyze the clinical, radiological, and functional outcomes of SPN for tibial shaft fractures treated in a semi-extended knee position. By focusing on fracture healing, knee functionality, and potential postoperative complications, this research seeks to contribute valuable insights into the efficacy and safety of this surgical technique.

## Materials and methods

This prospective study was conducted at Saveetha Medical College to evaluate the radiological and functional outcomes of patients diagnosed with tibial shaft fractures and treated with IMN using the suprapatellar approach. Patients aged 20 years or older, with closed fractures, segmental fractures, or fractures involving the proximal and middle third of the tibia, were included in the study. Exclusion criteria included pediatric fractures, patients over 70 years of age, significant comorbidities such as malignancies, refusal to participate, intra-articular fractures, and ipsilateral femoral fractures. The study was reviewed by Institutional Review Boards (IRBs) and approved by our institution's ethics committee.

Patients admitted to the study were assessed for surgical fitness, and those meeting the criteria underwent the procedure after informed consent was obtained. Patient demographics, surgical approach, intraoperative time, and blood loss were collected for all patients in the group. Operative and medical notes were reviewed to record intraoperative and postoperative complications during the hospital stay. Preoperative radiographs of ipsilateral knees were also taken to consider patellofemoral space as a prerequisite for SPN. The patients were followed for an average of 12 months to assess postoperative complications, fracture healing, and anterior knee pain. The primary outcome measures were intraoperative time and blood loss, while the secondary outcome measures included (1) the time to fracture union, as assessed by reviewing radiographs for radiological evidence of healing, and (2) the Lower Extremity Functional Scale (LEFS) and Visual Analog Scale (VAS) for knee pain.

Postoperative care included wound management with dressings on the second, fifth, and eighth postoperative days. Intravenous antibiotics were administered for one week, supplemented with supportive medications. Early knee and ankle range of motion exercises were initiated as soon as feasible. Sutures were removed in the second postoperative week. Clinical and radiological evaluations were conducted at one, three, six, and 12 months, assessing knee range of motion, fracture healing, and implant positioning.

A convenience sampling method was used, enrolling 20 patients in total. Statistical analysis was performed using SPSS software (version 28; IBM Corp., Armonk, NY, USA). Descriptive statistics were reported as the mean and standard deviation (SD), minimum-maximum, frequency, and percentage. Mann-Whitney U test was performed for continuous non-parametric data and the chi-square test for categorical variables to determine statistical significance. A p-value of <0.05 was considered to be statistically significant. 

## Results

The results of this prospective study provide a comprehensive overview of the patient demographics, intraoperative metrics, and fracture characteristics associated with SPN for tibial shaft fractures. The age distribution of the cohort showed that 70% of the patients were between 20 to 40 years old, indicating a higher prevalence of tibial shaft fractures in younger adults. The remaining 30% were aged between 41 to 60 years. The gender distribution revealed a predominance of male patients, who constituted 75% of the sample, while female patients made up 25%, highlighting a greater incidence of these fractures in men as shown in Table 1.

**Table 1:** Frequency distribution of age group and gender

Patient demographics	Frequency (n, %)
Age group	
20 to 40 years	14 (70%)
41 to 60 years	6 (30%)
Distribution of gender	
Male	15 (75%)
Female	5 (25%)

Distribution of fracture pattern

**Figure 1:**
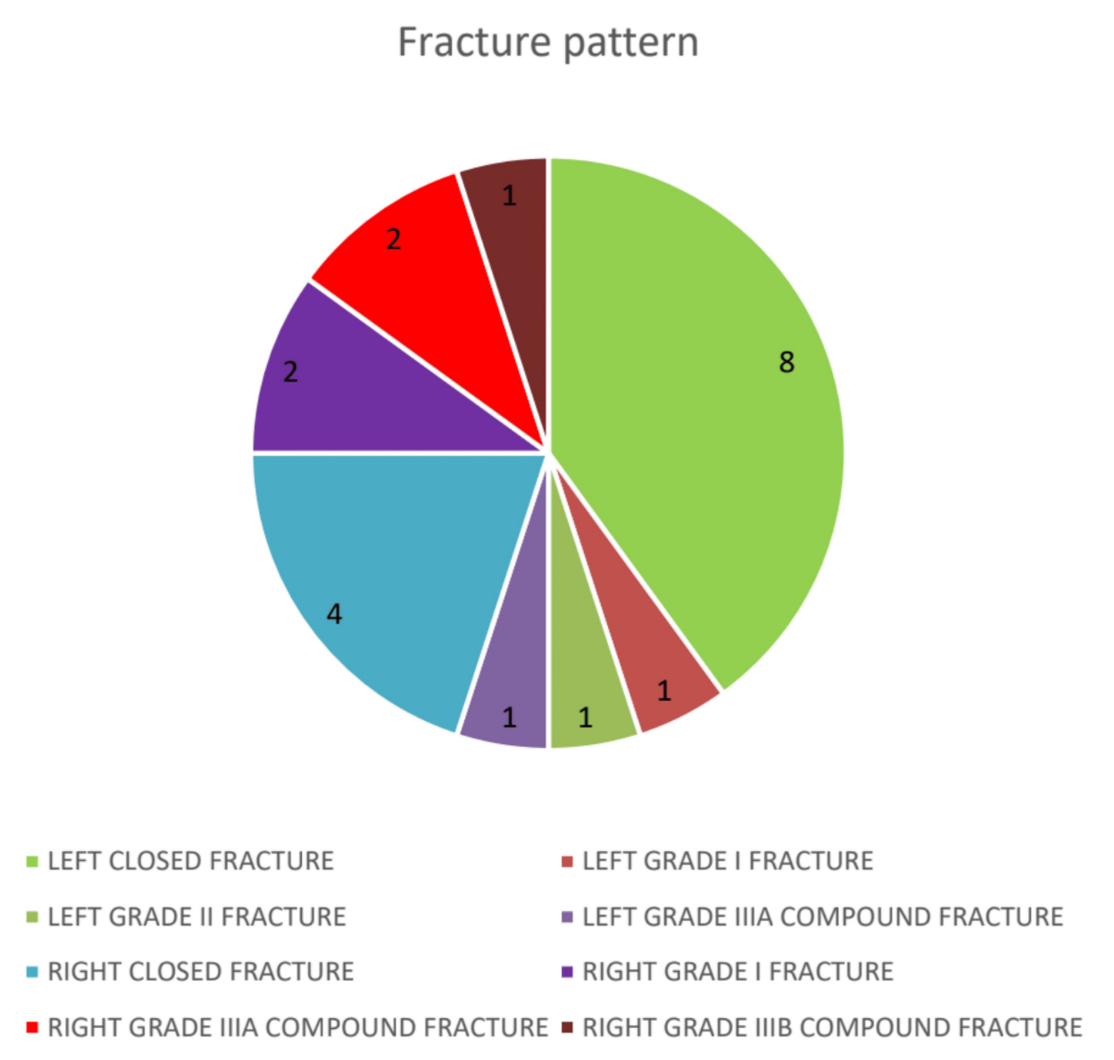
Frequency distribution of fracture patterns

The analysis of fracture patterns as per Gustilo Anderson classification in this study group revealed significant variations in both the type and laterality of fractures. Closed fractures were the most prevalent, with left-sided closed fractures constituting the largest proportion at 40% of the cases. This notable predominance of left-sided closed fractures may suggest potential biomechanical or environmental factors influencing the occurrence of fractures on this side. Right-sided closed fractures were less common, making up 20% of cases. Open fractures showed varied severity. Left-sided open fractures (Grades I, II, and IIIA) each accounted for 5% of cases, indicating a less frequent but diverse occurrence. Right-sided open fractures had higher incidences: Grade I and Grade IIIA fractures each represented 10%, while the more severe right-sided Grade IIIB accounted for 5% (Figure 1).

This distribution highlights that while closed fractures predominate, severe compound fractures, especially on the right side, are significant. The findings may relate to factors like handedness or activity type, with left-sided closed injuries being overall more common. Table 2 illustrates the detailed distribution and severity of these fracture types.

**Table 2:** Frequency distribution of side of injury and fracture pattern

Fracture pattern (%)	Laterality of injury (Gustilo Anderson classification)	Frequency (n, %)
Closed fracture	Left side	8 (40%)
	Right side	4 (20%)
Open fracture	Left side (Grade I)	1 (5%)
	Left side (Grade II)	1 (5%)
	Left side (Grade III A) - compound fracture	1 (5%)
	Right side (Grade I)	2 (10%)
	Right side (Grade III A)- compound fracture	2 (10%)
	Right side (Grade III B) - compound fracture	1 (5%)

**Figure 2:**
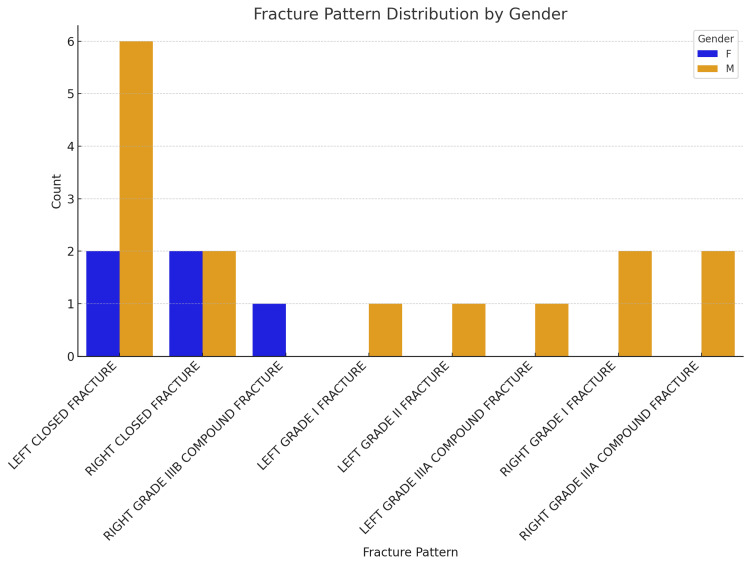
Fracture pattern distribution by gender

Among the 20 cases, the fracture patterns observed include a variety of types, with closed fractures being the most common across both genders. Compound fractures such as Grade IIIA and IIIB are seen predominantly in male patients, highlighting the severity and complexity of fractures within this group. Female patients, on the other hand, primarily exhibit closed fractures, with only one case involving a compound fracture. This data as shown in Figure 2 underscores a gender-based distribution, where males are more frequently affected and with more severe fracture patterns compared to females.

Surgical technique

The suprapatellar approach was used in all cases involved in the study. This method involves an entry point above the patella and is increasingly favored for its potential benefits, such as improved alignment, reduced soft tissue damage, and better patient outcomes, particularly in complex fractures or when minimizing joint disruption is a priority.

**Figure 3:**
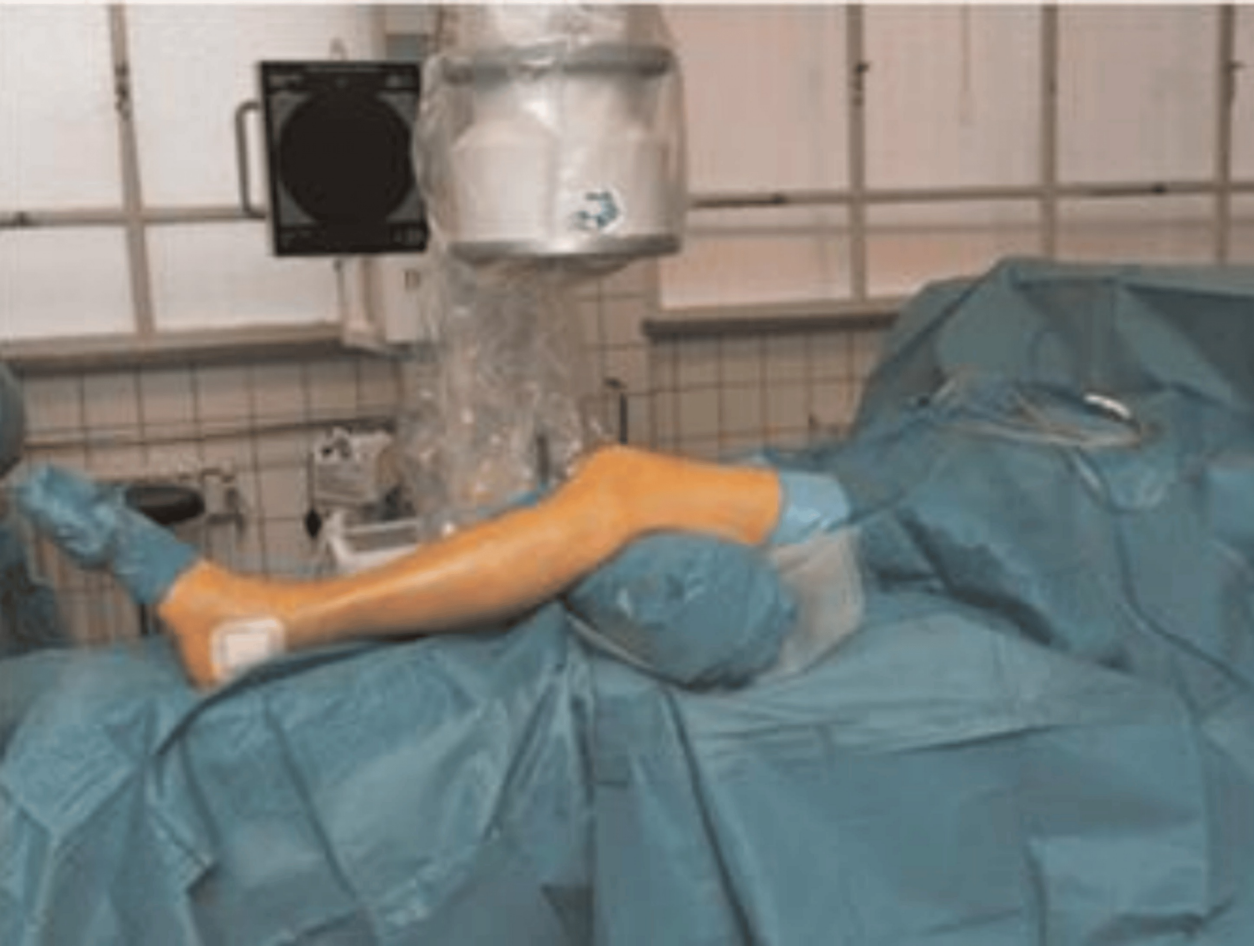
Position of the patient and the image intensifier

**Figure 4:**
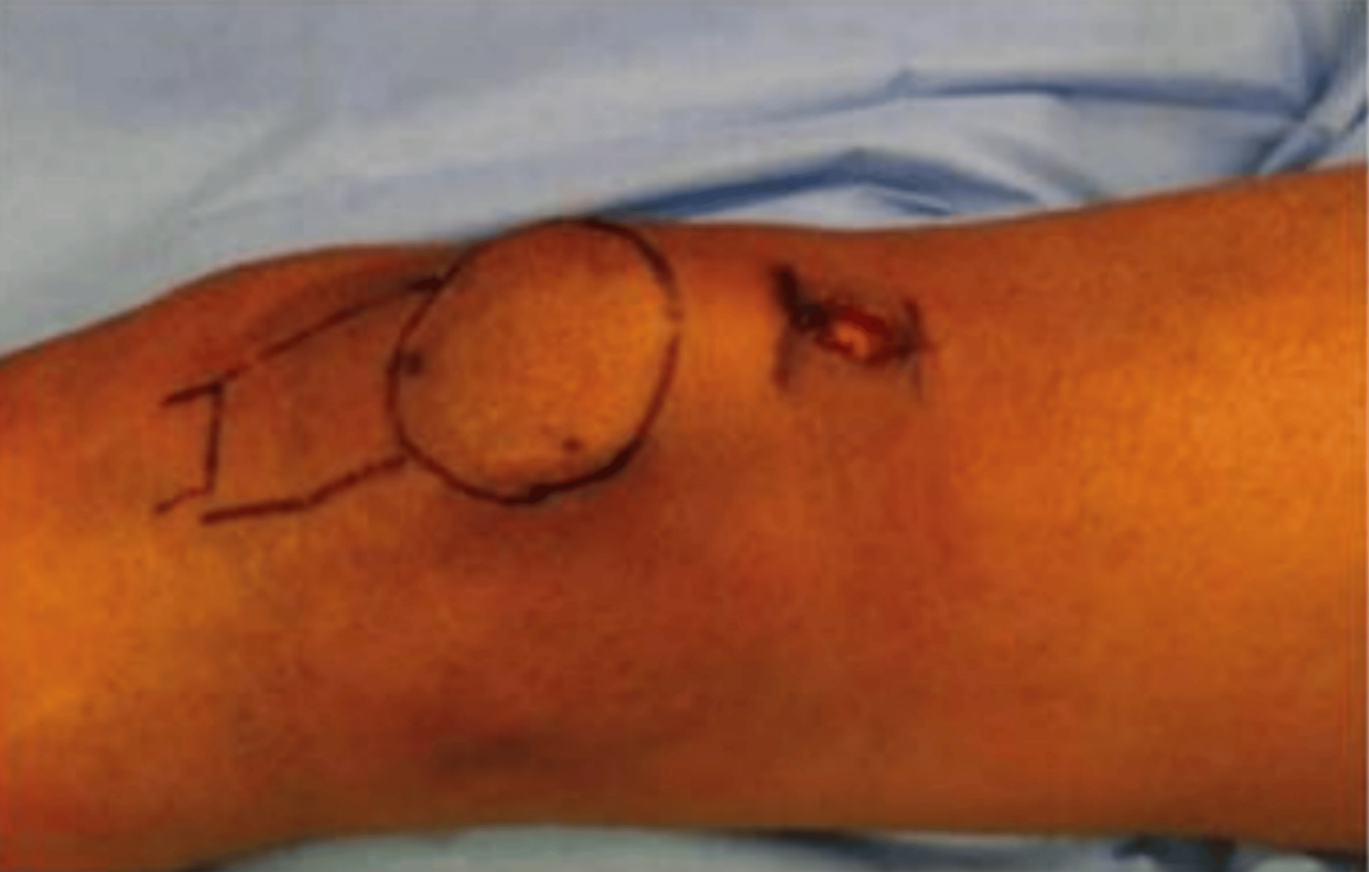
Skin incision for suprapatellar approach

**Figure 5:**
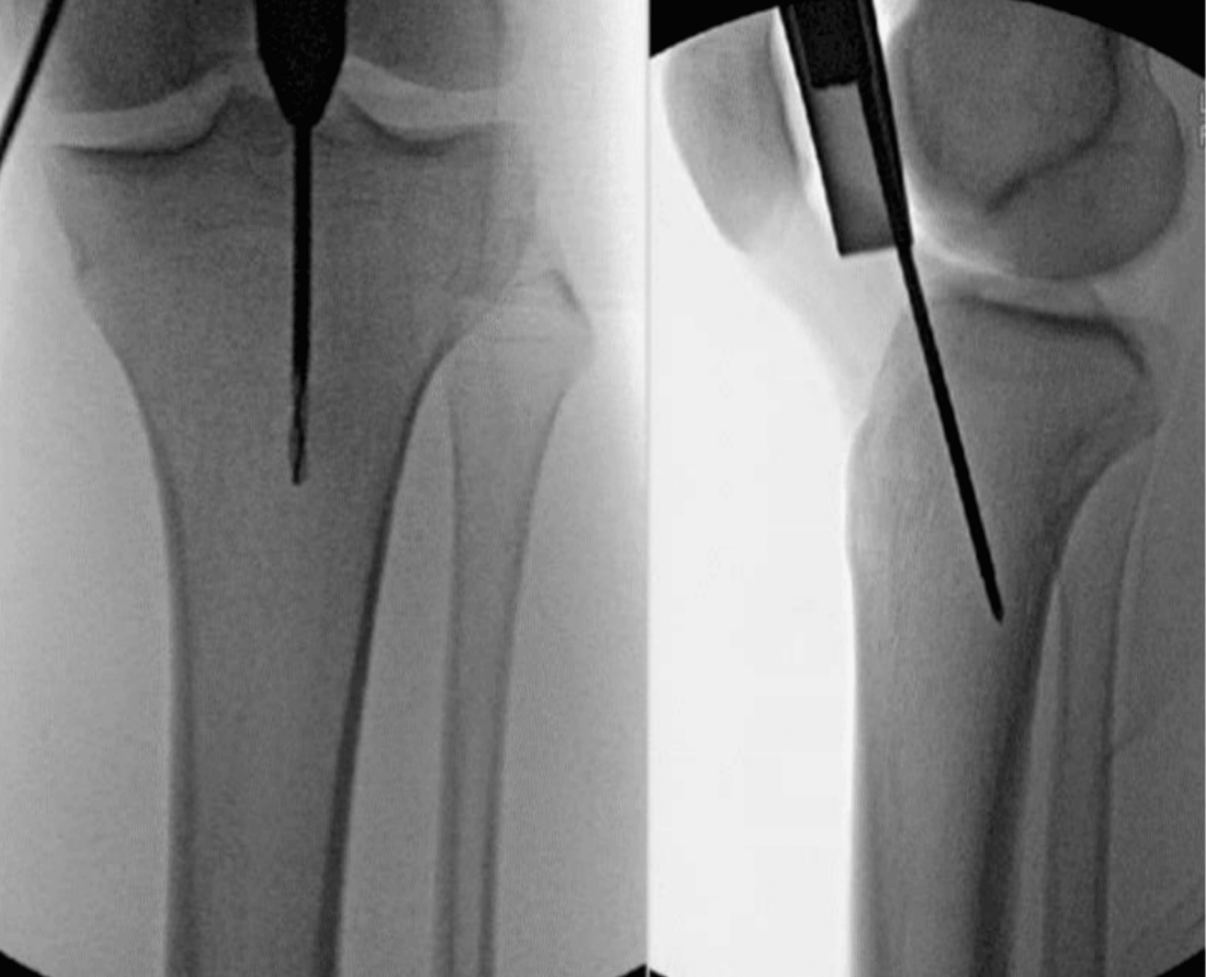
Position of the guide wire under image intensifier

In this study, the surgical approach involved positioning patients supine with the knee in a semi-extended position, supported by a bolster, under strict aseptic conditions as shown in Figure 3. A proximal skin incision above the patella was made (Figure 4), followed by careful separation of subcutaneous tissues and splitting of the quadriceps muscle. A trocar was inserted retro-patellarly to guide the cannula to the tibia, protecting intra-articular structures during reaming. The guide wire was positioned using an image intensifier (Figure 5), ensuring precise placement relative to the tibial spine. Entry to the medullary canal was achieved with an awl, followed by serial reaming. A nail was then inserted, with proximal and distal locking performed to stabilize the fracture, verified with image guidance, before wound closure.

Duration of surgery and blood loss

The distribution of surgery durations among the study's subjects is outlined in Table 3, showcasing a range of times with various frequencies. The most common surgery duration recorded was 90 minutes, with four subjects (20%) falling into this category. This suggests that 90 minutes is a typical benchmark for the majority of these procedures. The next most frequent durations were 60 and 100 minutes, each accounting for three subjects (15%). These durations indicate that a significant portion of the surgeries also tended to fall within these time frames, reflecting a secondary peak in distribution. The rest of the surgical time recorded were varied, from the shortest recorded at 60 minutes to the longest at 112 minutes. This highlights the variability in surgical procedure durations, possibly influenced by factors such as the complexity of the case or surgical approach. 

**Table 3:** Frequency distribution of surgery time

Surgery time (minutes)	Frequency (n, %)
60	3 (15%)
62	1 (5%)
65	1 (5%)
70	2 (10%)
80	1 (5%)
89	1 (5%)
90	4 (20%)
95	1 (5%)
100	3 (15%)
110	1 (5%)
112	1 (5%)

Table 4 shows the distribution of blood loss during surgery, with the most common amount being 50 mL, observed in six subjects (30%). Blood loss of 40 ml and 60 ml were seen in 3 and 5 subjects, respectively, indicating that most procedures experienced relatively minimal blood loss. Less frequent amounts including blood loss from 100 mL to 200 mL were each seen in one subject (5% each). The data highlights that while the majority of surgeries had blood loss at or below 60 mL, there were occasional cases with higher losses, reaching up to a maximum of 200 mL. 

**Table 4:** Frequency distribution of blood loss

Blood loss (ml)	Frequency (n, %)
40	3 (15%)
50	6 (30%)
60	5 (25%)
100	1 (5%)
120	1 (5%)
150	1 (5%)
180	1 (5%)
190	1 (5%)
200	1 (5%)

Fracture union time and outcome comparison

The table depicting the frequency distribution of the time of union in weeks (Table 5) shows that the majority of subjects (45%) experienced union at 12 weeks, making it the most common time frame. A smaller proportion, 25%, had a union at 14 weeks, while 20% had a union at 16 weeks. The longest time frame for the union was greater than 28 weeks, observed in only two subjects (10%), indicating that extended recovery periods were less common.

**Table 5:** Frequency distribution of time of union of fractures

Time of union (weeks)	Frequency (n, %)
12	9 (45%)
14	5 (25%)
16	4 (20%)
>28	2 (10%)

The frequency distribution of the LEFS scores shows that the highest proportion of subjects (40%) achieved a score of 80, suggesting favorable functional outcomes for these individuals (Table 6). A score of 76 was seen in 20% of subjects, while 10% scored 70 and 72, respectively. Lower scores such as 66, 68, 75, and 78 were observed in 5% of subjects each, indicating varying levels of lower extremity function within the study group. The VAS score outcomes indicate that the majority of patients (60%) reported mild pain levels, with scores of 2 or 3, while fewer patients experienced moderate to higher pain levels, as reflected by scores of 4 (15%) and 5 (10%). This suggests generally favorable pain management outcomes post-surgery. 

**Table 6:** Frequency distribution of LEFS and VAS score LEFS: Lower Extremity Functional Scale; VAS: Visual Analog Scale

LEFS score	Frequency (n, %)	VAS score	Frequency (n, %)
66	1 (5%)	1	3 (15%)
68	1 (5%)	2	6 (30%)
70	2 (10%)	3	6 (30%)
72	2 (10%)	4	3 (15%)
75	1 (5%)	5	2 (10%)
76	4 (20%)		
78	1 (5%)		
80	8 (40%)		

Regarding complications, the data indicates that most subjects (85%) did not experience any complications or require any additional intervention, which highlights a positive outcome for the majority. However, 15% (n=3) of subjects each had delayed union, infected non-union, and proximal screw infection. This demonstrates that while complications were generally rare, they were still present in a small number of cases. 

Summary of comparison among various factors

Table 7 shows a summary of the mean and SD for surgery time, blood loss, time of union, follow-up duration, and LEFS score. Surgery times ranged from a minimum of 60 minutes to a maximum of 112 minutes, with a mean of 83.5 minutes and an SD of 17.08. Blood loss varied significantly, from 40 mL to 200 mL, with a mean of 91 mL and an SD of 57.48, reflecting a wide range in procedural blood loss. The time of union had a mean of 12.1 weeks (SD: 4.42), and the follow-up duration ranged from seven to 18 months, with a mean of 11.35 months (SD: 2.11).

**Table 7:** Summary statistics of clinical parameters

Parameters	N	Minimum	Maximum	Mean	Standard deviation
Surgery time (mins)	20	60	112	83.5	17.080
Blood loss (mL)	20	40	200	91.00	57.482
Time of union	20	0	16	12.10	4.424
Follow-up duration	20	7	18	11.35	2.110
LEFS score	20	66	80	75.75	4.587

**Figure 6:**
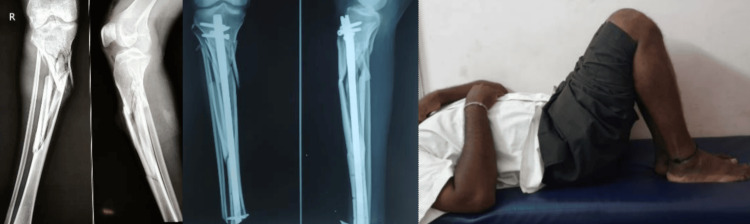
Pre- and postoperative follow-up image of the tibial fracture

**Figure 7:**
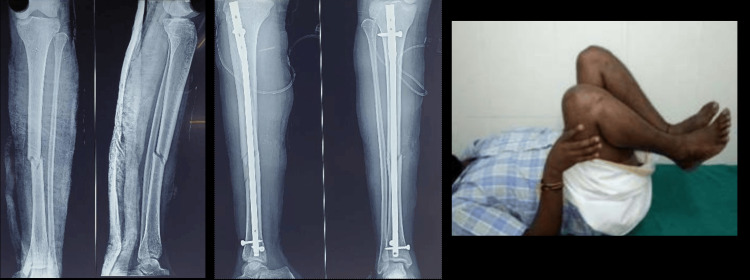
Pre- and postoperative follow-up image of the tibial fracture

The LEFS scores ranged between 66 and 80, with a mean score of 75.75 (SD: 4.59). The 95% confidence intervals depicted indicate variability across these parameters, while the odds ratio comparison shows that the majority of subjects had no complications. Two example illustrations of pre- and postoperative radiographs along with the range motion from the study have been shown in Figures 6, 7.

## Discussion

This prospective study provides valuable insights into the demographic characteristics, fracture patterns, surgical metrics, and outcomes associated with SPN for tibial shaft fractures. The findings align with existing literature and contribute to the ongoing discourse on optimal surgical approaches for these injuries. The predominance of younger adults (70% aged 20-40 years) and males (75%) in our cohort is consistent with global epidemiological trends. Tibial shaft fractures are commonly associated with high-energy trauma, such as motor vehicle accidents, which are more frequent among younger males [14]. The higher incidence of left-sided closed fractures (40%) observed may be influenced by factors such as handedness and typical mechanisms of injury, though further research is needed to elucidate these associations.

The traditional, IP nailing method requires flexion or hyperflexion of the knee. A significant drawback of the IP approach is the high rate of anterior knee pain, affecting 10-80% of patients. The suprapatellar approach, by avoiding the patellar tendon, reduces the incidence of anterior knee pain. Multiple studies have reported lower postoperative pain scores with the suprapatellar approach, enhancing patient satisfaction and improving functional outcomes [15].

The suprapatellar approach was utilized in 75% of cases, reflecting its growing acceptance in orthopedic practice. This technique offers advantages in fracture alignment and reduced soft tissue disruption [16]. However, the primary challenge in utilizing the suprapatellar approach for tibial nailing is the potential risk of damage to the chondral surface of the patella, the knee joint, and the anterior horn of the meniscus. Multiple studies have shown no significant differences between the suprapatellar and IP approaches regarding postoperative pain, knee range of motion, or knee functional outcomes [8,17,18]. Conversely, Gaines et al. reported a reduced risk of articular structure damage with the SPN technique [19]. Additionally, MacDonald et al. demonstrated that the suprapatellar approach for antegrade tibial nailing is associated with less postoperative anterior knee pain compared to the IP approach [20]. These findings suggest that the suprapatellar approach may provide advantages in minimizing knee joint complications and enhancing patient comfort post-surgery.

Our study found that the suprapatellar approach had a longer mean surgery time (95.5 minutes) compared to the parapatellar approach (65.5 minutes). However, it was associated with significantly lower mean blood loss (50.0 mL vs. 152.5 mL), suggesting a trade-off between operative duration and intraoperative blood loss. By avoiding manipulation near the patellar tendon and limiting soft tissue dissection, the suprapatellar approach decreases the risk of complications such as tendonitis or patellar tendinopathy, which are more common in the IP technique due to the tendon handling and flexion positioning involved [15]. The majority of patients (45%) achieved fracture union within 12 weeks, aligning with standard healing timelines for IMN of tibial fractures. Delayed unions were observed in a minority of cases (10%), which is comparable to rates reported in other studies [14]. Functional outcomes, as measured by the LEFS, were favorable, with 40% of patients attaining the maximum score of 80. This underscores the efficacy of the suprapatellar approach in facilitating satisfactory postoperative function. Complication rates were low, with 85% of patients experiencing no postoperative issues. The incidence of delayed union, infected non-union, and proximal screw infection (each at 5%) is consistent with existing literature on IMN complications [14,16]. These findings suggest that the suprapatellar approach does not increase the risk of such complications and may offer a safe alternative to traditional methods.

Our findings align with several studies that highlight the benefits of the suprapatellar approach for tibial nailing. For example, Avilucea et al. conducted a study on suprapatellar IMN and found that this technique improved alignment and reduced malalignment rates compared to the IP approach, with no significant differences in functional scores or complications between the two methods [13]. Similarly, Sun et al. conducted a large cohort study and observed that the suprapatellar approach led to significantly lower postoperative anterior knee pain and higher Lysholm knee scores at various follow-up intervals, indicating a functional advantage over the IP method [21]. These findings support the suprapatellar approach as a viable alternative for tibial fractures, particularly in cases where patient comfort and functional outcomes are priorities.

Additionally, Çiçekli et al. studied SPN across different tibial fracture levels and concluded that the suprapatellar approach was effective for extra-articular tibial fractures at all locations, with satisfactory healing times and low pain scores (VAS score ≤3 in 84.5% of patients) [22]. This conclusion is reinforced by Gao et al.'s meta-analysis, which found that the suprapatellar approach was associated with reduced blood loss, decreased postoperative pain, shorter fluoroscopy times, and improved knee function scores, without an increase in postoperative complications compared to the IP approach [23]. These studies demonstrate that the suprapatellar approach offers consistent advantages across various fracture patterns, contributing to improved patient outcomes and a smoother postoperative recovery process.

The findings of this study support the adoption of the suprapatellar approach for IMN of tibial shaft fractures, particularly in cases where minimizing blood loss and optimizing postoperative function are priorities. While the longer operative time associated with this technique warrants consideration, the overall benefits in terms of reduced complications and favorable functional outcomes may justify its use. This study's limitations include a relatively small sample size and the lack of a randomized control group. Future research with larger, randomized cohorts is necessary to further validate these findings and to explore the long-term outcomes associated with the suprapatellar approach. Additionally, investigations into patient-reported outcomes and quality-of-life measures would provide a more comprehensive understanding of the benefits and potential drawbacks of this surgical technique.

## Conclusions

This study supports the suprapatellar approach for IMN of tibial shaft fractures as a favorable technique, demonstrating multiple advantages in patient outcomes and surgical efficiency. The semi-extended position allows for improved control of proximal fracture fragments, particularly beneficial for proximal tibial fractures, and facilitates better fracture reduction, alignment, and imaging during surgery. With significantly reduced total blood loss, less postoperative knee pain, and improved functional outcomes, this approach minimizes common complications associated with the IP method. 

Furthermore, the use of a specialized cannula system at the entry point reduces the risk of intra-articular damage, enhancing safety during the procedure. The suprapatellar approach, therefore, offers a practical and effective solution for closed tibial fractures, with fewer risks of anterior knee pain and malunion, reduced fluoroscopy time, and early rehabilitation benefits. This technique may serve as an ideal alternative to the conventional IP approach, with implications for minimizing early arthritis risks. Further long-term, large-scale studies are warranted to validate these findings and support ongoing innovation in orthopedic trauma management.
